# Urinary metabolomic profiling to identify the impact of the increased intake of whole grains, vegetables, and fruits in healthy adolescents

**DOI:** 10.1038/s41430-026-01703-8

**Published:** 2026-07-29

**Authors:** Mi-Ri Gwon, Jae Hwa Lee, Duk-Hee Lee, Yoo Kyoung Park, Soo-Jin Park, Ji-Won Lee, Hae Won Lee, Sook Jin Seong, Young-Ran Yoon

**Affiliations:** 1 School of Medicine, Kyungpook National University and Department of Clinical Pharmacology and Therapeutics, Kyungpook National University Hospital, Daegu, Republic of Korea; 2 Clinical Omics Institute, School of Medicine, Kyungpook National University, Daegu, Republic of Korea; 3 Department of Preventive Medicine, School of Medicine, Kyungpook National University, Daegu, Republic of Korea; 4 Department of Medical Nutrition, Graduate School of East-West Medical Science, Kyung Hee University, Yongin, Republic of Korea; 5 Department of Histology and Anatomy, College of Korean Medicine, Daegu Haany University, Gyeongsan, Republic of Korea; 6 Department of Family Medicine, Severance Hospital, Yonsei University College of Medicine, Seoul, Republic of Korea

**Keywords:** Nutrition, Public health

## Abstract

**Background:**

A plant-based diet is reportedly effective in preventing chronic and metabolic diseases, primarily because of the antioxidant properties of phytochemicals present in vegetables and fruits. However, systematic metabolomics studies investigating the changes in metabolic phenotypes resulting from plant-based diets in adolescents are lacking. Therefore, this study aimed to examine alterations in urinary metabolomic profiles associated with a plant-based diet to explore its beneficial metabolic effects.

**Methods:**

Eighteen healthy Korean adolescents participated in an 8-week intervention, in which they modified their dietary patterns to primarily include whole grains, vegetables, and fruits. Urine samples were analyzed using gas chromatography–mass spectrometry, and multivariate analysis was performed to identify statistically significant metabolite alterations following the plant-based dietary intervention.

**Results:**

The plant-based diet significantly reduced body weight and body fat mass but increased plasma biological antioxidant potential. Metabolomic profiling revealed a clear distinction between samples collected before and after the 8-week intervention, with R^2^Y and Q^2^ values for the permuted models at 0.906 and 0.635, respectively. Key metabolite changes included increases in L-phenylalanine, L-tyrosine, glutathione, succinic acid, and tartaric acid as well as decreases in L-leucine, glycine, and indoleacetic acid. These changes suggest enhancements of antioxidant capacity, detoxification pathways, and mitochondrial function.

**Conclusion:**

This nutritional metabolomics study provides mechanistic insights into the metabolic changes associated with a plant-based diet. Its findings support the integration of plant-based meals into school settings to promote the metabolic health of adolescents and mitigate the risk of chronic disease progression.

## Introduction

In modern society, concerns regarding long-term health risks, such as obesity and chronic metabolic diseases, are increasing as daily vegetable and fruit intake among adolescents declines worldwide, including in South Korea [1]. This trend exacerbates national health issues and socioeconomic burdens. A plant-based diet has been proposed to address these concerns. This diet emphasizes whole grain, vegetable, fruit, legume, nut, and vegetable oil consumption and may include dairy products and/or eggs. It has garnered significant attention owing to its numerous health benefits. It is characterized by lower saturated fat, cholesterol, and animal protein levels, while providing higher fiber, magnesium, and antioxidant (e.g., vitamin C, vitamin E, carotenoids, and phytochemicals) levels [2]. Lipid-soluble antioxidants (*e.g*., β-carotene and vitamin E) reduce cardiovascular and chronic kidney disease risks [3, 4], while vitamin C and polyphenols enhance the immune system and lower cancer risk [5–7]. Epidemiological and clinical studies have consistently reported positive health outcomes associated with plant-based diets, such as improved weight management, better glycemic and lipid profiles, and reduced chronic inflammation. A recent prospective study conducted in the United Kingdom found that adherence to a healthful plant-based diet was significantly associated with a reduced risk of mortality, cardiovascular disease, and cancer [8]. The World Cancer Research Fund recommends consuming ≥400 g/day of diverse whole grains, vegetables, and fruits to decrease the overall cancer incidence [9].

A well-planned plant-based diet can satisfy the nutritional needs of adolescents and promote normal growth and development [10]. Adolescents who follow a plant-based diet typically consume more vegetables, fruits, fiber, folate, vitamin A, and vitamin C [11, 12]. A recent meta-analysis indicated that girls with higher fiber and monounsaturated fat intakes are less likely to experience early menarche [13], a factor associated with increased hormone-related cancer, cardiovascular disease, and overall mortality risks. Therefore, incorporating a plant-based diet into school systems is an effective strategy for establishing healthy nutritional intake during adolescence, supporting healthy development, and alleviating the risk of chronic diseases later in life. Although numerous studies have investigated the health benefits of a plant-based diet, evidence regarding its metabolic effects on adolescents remains limited.

Untargeted metabolomics, which involves the comprehensive profiling of metabolites in biological samples, offers a system-level approach to understanding global metabolic regulation within an organism. This method facilitates the correlation of metabolomic data with demographic and anthropometric information, dietary habits, and disease states [14]. Nutritional metabolomics has recently unearthed new strategies for exploring the relationships among diet, physiological factors, and metabolism. Despite the growing interest in this field, the impact of plant-based diets on metabolomic profiles in adolescents remains unclear. To address this knowledge gap, we investigated the alterations in urinary metabolomic profiles associated with a plant-based diet in healthy Korean adolescents.

## Materials and methods

### Study design and participants

This 8-week intervention was approved by the Institutional Review Board of Kyungpook National University Hospital (KNUH2011-08-015) and registered with the Clinical Research Information Service (https://cris.nih.go.kr; KCT0002372). Participants were recruited from a single high school (Daegu Seobu High School, Daegu, Republic of Korea). Written informed consent was obtained from all participants and their legal guardians after a full explanation of the study objectives and procedures, in accordance with the Declaration of Helsinki and the approved Institutional Review Board protocol. Healthy Korean adolescents aged 16–18 years with no significant medical history were enrolled. The exclusion criteria included: (1) regular whole grain, vegetable, and fruit intake; (2) medication use; (3) chronic disease; (4) alcohol consumption at least once a week; or (5) smoking. Twenty-five participants were recruited; however, two were lost to follow-up, and five withdrew, resulting in 18 completing the 8-week study.

### Blood clinical laboratory tests

Participants visited the Seo-gu District Public Health Center (Daegu, Republic of Korea) under school coordination in the morning, both before and after the intervention. Fasting blood samples were collected by a trained clinical technician into EDTA tubes for hematologic parameters and serum-separating tubes for biochemical analyses. EDTA tubes were gently inverted several times to ensure proper mixing of the anticoagulant with the blood, whereas serum-separating tubes were kept upright to allow clot formation. Subsequently, all samples were centrifuged at 3000 *rpm* for 10 min at 4 °C. Plasma total cholesterol, triglyceride, blood cell count, and mineral levels were measured at GC Lab (Yongin, Republic of Korea) using enzymatic colorimetric methods. Systemic oxidative stress was assessed based on plasma hydroperoxide levels using the diacron-reactive oxygen metabolites (d-ROMs) test (Diacron S.R.L., Grosseto, Italy). Antioxidant potential was evaluated using the biological antioxidant potential (BAP) test, measuring plasma antioxidants by reducing ferric ions to ferrous ions. The oxidative stress index (OSI) was determined by calculating the d-ROMs/BAP ratio [15].

### Dietary intervention and assessment

Participants followed a plant-based diet encompassing: (1) brown rice-based whole grains, (2) 8–10 servings of vegetables (560–700 g), (3) one serving of fruit (100 g), (4) three types of protein-rich plant-based side dishes (e.g., legumes or tofu), and (5) a fresh apple. Meat, fish, and dairy products were permitted once a week to minimize potential health risks associated with a sudden dietary change. A registered dietitian formulated the diet, and a cooking session focusing on the plant-based diet was organized for parents, along with educational sessions to ensure adherence to the diet outside of school and on weekends. A detailed weekly meal plan representing the plant-based diet is provided in Supplementary Table 1.

Nutrient intake was assessed using 93 questions from the Food Frequency Questionnaire for Korean Adolescents (FFQ-A) [16] in conjunction with the Korean Food Composition Table [17]. Based on the Korea National Health and Nutrition Examination Survey [18], the FFQ-A was designed to reflect the dietary patterns of this demographic. The FFQ-A was administered before and after the intervention to evaluate compliance and dietary changes. Selected food groups from the FFQ-A are presented in Supplementary Table 2. The questionnaire quantifies the frequency of food consumption across nine categories, ranging from “three times per day” to “rarely eat”, based on three serving size units for the cooked food: 0.5 standard serving (count), 1 standard serving (count), and 1.5 standard servings (count). The FFQ-A exclusively includes β-carotene values from vegetables and fruits. Nutrient intake was calculated using CAN-Pro 3.0 (Korea Institute of Nutrition, 2005).

### Urinary metabolomic profiling

Urine was selected as the metabolomic matrix because it provides a noninvasive and participant-friendly alternative to blood sampling. This biofluid is enriched in water-soluble metabolites involved in antioxidant and energy metabolism, allowing sensitive detection of low-abundance compounds [19]. First-morning urine samples, collected together with blood samples, were immediately frozen at –70 °C prior to analysis. Pooled quality control (QC) samples were prepared by mixing equal volumes of all urine samples. The chemicals and reagents used in metabolomic profiling are described in the supplementary information. To remove urea, urease (16 units) was added to urine (100 μL), which was subsequently incubated at 37 °C for 30 min. This enzymatic step was performed to prevent excessive urea from reacting with the derivatizing reagent and forming abundant trimethylsilyl-urea derivatives, which could suppress the ionization of low-abundance metabolites by consuming derivatization reagents and contaminating the GC column. Therefore, urea removal improves derivatization efficiency and enables more accurate and reproducible profiling of biologically relevant metabolites in untargeted analyses [20, 21]. After adding cold methanol (400 μL) and 10 μL of the internal standard (1000 *ppm*), the samples were vortexed for 5 min and centrifuged at 13,200 *rpm* for 10 min at 4 °C. The supernatant (200 µL) was dried under nitrogen at 45 °C. The residue was dissolved in methoxylamine hydrochloride (50 μL) to prevent ring structure formation from reducing sugars and protect aldehydes and ketones. The solution was subsequently vortexed and incubated for 90 min at 30 °C. Thereafter, a silylation reagent (50 μL) was added and the resulting mixture incubated at 37 °C for 1 h to enable derivatization. The final solution was transferred to an autosampler vial and analyzed via gas chromatography–mass spectrometer (GC-MS).

For metabolomic profiling, an Agilent 7890 A GC coupled with a 5975 C MS (Agilent Technologies, Palo Alto, CA) was utilized. An HP-5ms fused silica capillary column (5% phenyl-95% dimethylpolysiloxane, 30 m × 0.25 mm × 0.25 μm; J&W Scientific, Folsom, CA) was used, with helium (1.235 mL/min) as the carrier gas. The injector and ion source were maintained at 350 and 230 °C, respectively. The samples were injected in 2-μL volumes using a 5:1 split ratio. The column oven was initially maintained at 70 °C for 0.1 min, subsequently increased to 310 °C at 5 °C/min, and finally held for 2 min. The MS operated in electron ionization mode at 70 eV, acquiring data in full scan mode within an *m/z* range of 50–550.

### Data analysis

Raw data were converted to netCDF format and processed using XCMS for deconvolution and peak detection. Systemic variation was eliminated using quantile normalization, and the internal standard was utilized for normalization and peak alignment. The normalized feature table was exported to SIMCA 18 (Umetrics, Umeå, Sweden) and scaled for unit variance. Multivariate analyses, principal component analysis (PCA), and orthogonal partial least squares-discriminant analysis (OPLS-DA), were conducted. The OPLS-DA model was validated using a permutation test with 100 iterations, evaluating goodness of fit (R^2^X) and predictive ability (Q^2^). Features with a variable importance in projection (VIP) value ≥ 1.0 were considered significant for group discrimination. Kyoto Encyclopedia of Genes and Genomes (KEGG)-based pathway analysis was performed using MetaboAnalyst 6.0 (https://www.metaboanalyst.ca). Correlation analysis was conducted in R 4.3.1 (version 4.3.1, https://www.r-project.org) and visualized with Cytoscape 3.4.0.

### Metabolite identification

Components exhibiting matching rates ≥85% were screened against the Wiley 10th/NIST 2012 Library (John Wiley & Sons, Inc., Hoboken, NJ). Features with a VIP value ≥ 1.0 were putatively annotated using the Human Metabolome Database (http://www.hmdb.ca), MassBank (http://www.massbank.jp), METLIN (https://metlin.scripps.edu), the Fiehn Library (http://fiehnlab.ucdavis.edu/Metabolite-Library-2007), the KEGG (http://www.genome.jp/kegg), and PubChem (http://pubchem.ncbi.nlm.nih.gov) [22]. The retention times and MS spectra of putatively annotated metabolites were compared with those of authentic standards.

### Statistical analysis

Statistical analyses were performed in R. Parametric data were analyzed using a paired *t* test and are presented as the mean (standard deviation). Nonparametric data were analyzed using Wilcoxon signed-rank tests and are expressed as the median [interquartile range]. Statistical significance was established at *p* < 0.05.

## Results

Table 1 summarizes the demographic and anthropometric characteristics of participants as well as their clinical parameters following the 8-week intervention. Participants experienced significant reductions in body weight (3.4 kg, *p* < 0.001), body mass index (1.3 kg/m², *p* < 0.001), and body fat mass (4.3 kg, *p* < 0.001). Although certain variables exhibited changes, all measurements remained within normal physiological reference ranges.

**Table 1:** Changes in demographic and anthropometric parameters before and after the plant-based dietary intervention.

Variable	Baseline	After 8 weeks	*p* value
**Age, years**	16.2 (0.4)	–	
**Boy/Girl,** ***n***	8/10	–	
**Anthropometry**			
**Height, cm**	167.2 (9.2)	167.2 (9.4)	0.098
**Weight, kg**	65.8 (14.3)	62.4 (14.0)	<0.001^*^
**Body mass index, kg/m**^**2**^	23.4 (3.8)	22.1 (3.8)	<0.001^*^
**Body fat mass, kg**	17.2 (7.1)	12.9 [8.7]	<0.001^*^
**Body fat, %**	25.5 (6.6)	23.5 (8.4)	0.005^*^
**Body muscle mass, kg**	44.9 (8.6)	43.8 (8.4)	0.003^†^
**Body mineral, kg**	3.8[0.8]	3.3[1.1]	<0.001^†^
**Body protein, kg**	9.7[3.1]	10.1[3.3]	0.396
**Blood lipids**			
**Total cholesterol, mg/dL**	152[31.5]	163[58.0]	0.744
**Triglycerides, mg/dL**	95[63.8]	72[34.3]	0.695
**Complete blood cell count**			
**Hemoglobin, g/dL**	14.4 (1.6)	15.1 (0.9)	0.112
**Hematocrit, %**	41.2 (4.6)	42.6 (2.2)	0.223
**Red blood cell, 10**^**6**^**/**μ**L**	4.7 (0.5)	4.8 (0.4)	0.334
**White blood cell, 10**^**3**^**/ L**	6.8 (1.3)	6.1 (1.2)	0.138
**Platelet, 10**^**3**^**/**μ**L**	280.3 (45.6)	283.6 (50.2)	0.850
**Zinc**, μ**g/dL**	119.6 (18.4)	89.5 (9.1)	<0.001^*^
**Iron**, μ**g/dL**	81.5 (35.2)	107.9 (43.3)	0.075
**Calcium, mg/dL**	9.5 (0.3)	9.7 (0.3)	0.044^*^
**Magnesium, mg/dL**	2.2 (0.1)	2.2[0.2]	0.138

Statistical comparisons were performed using a paired *t* test^*^ when the normality assumption was met; otherwise, the Wilcoxon signed-rank test^†^ was employed.

Data are presented as the mean (standard deviation) for normally distributed variables or as the median [interquartile range] for non-normally distributed variables.

The *p* < 0.05 considered statistically significant.

During the intervention, total energy intake decreased by approximately 4,186 kJ (1000 kcal). Carbohydrate, protein, and fat distributions shifted from 66%, 21%, and 13% of total energy intake to 84%, 10%, and 6%, respectively (Table 2). Notably, cholesterol intake significantly decreased (*p* < 0.001). The proportion of carbohydrates increased to 84% of total energy intake, consistent with the characteristics of a plant-based diet emphasizing whole grains, vegetables, and fruits. Additionally, plant- and animal-derived calcium both decreased (*p* = 0.031 and *p* < 0.001, respectively), whereas iron decreased mainly from animal sources (*p* < 0.001). The intakes of phosphorus (*p* < 0.001) and zinc (*p* = 0.005) also declined following the intervention, primarily due to the reduction in animal-based food sources. Conversely, plant-based iron intake remained relatively stable. As shown in Table 2, the daily intake of eight vitamins—vitamin A, retinol, vitamin C, vitamin E, and B-complex vitamins (B1, B2, B6, and niacin)—significantly decreased (all *p* < 0.05).

**Table 2:** Comparison of daily energy and nutrient intakes estimated from a food frequency questionnaire and 24-h recall in Korean adolescents before and after the plant-based dietary intervention.

Variable	Baseline	After 8 weeks	*p* value
**Energy, kJ**	11675.2 (3128.2)	7711.4 (3471.9)	0.002^*^
**Carbohydrate, % of energy**	67.4 (6.5)	86.1 [4.9]	<0.001^*^
**Carbohydrate, g**	463.3 (107.9)	388.1 (181.5)	0.124
**Protein, % of energy**	12.7 (2.0)	10.5 (1.9)	<0.001^*^
**Plant-source protein, g**	54.2 (16.1)	45.8 (25.0)	0.117
**Animal-source protein, g**	35.3 (20.3)	1.5 [2.0]	<0.001^*^
**Fat, % of energy**	20.4 (5.7)	5 [2.5]	<0.001^*^
**Plant-source fat, g**	31.2 (18.8)	9.5 (4.9)	<0.001^*^
**Animal-source fat, g**	34.2 (17.8)	1.4 [1.6]	<0.001^*^
**Cholesterol, mg**	260.9[303.0]	21.1 [30.0]	<0.001^†^
**Fiber, g**	19.8[24.1]	16.5 [12.2]	0.089
**Calcium**			
**Plant-source calcium, mg**	392.7 (173.9)	249.9 [144.5]	0.031^*^
**Animal-source calcium, mg**	288.3 (178.3)	3.3 [6.4]	<0.001^*^
**Phosphorus, mg**	1308.1 (451.5)	698.7 (302.1)	<0.001^*^
**Iron**			
**Plant-source iron, mg**	13.8 (5.4)	12.3 [7.0]	0.486
**Animal-source iron, mg**	3.3 (2.3)	0.2 [0.2]	<0.001^*^
**Sodium, mg**	3555.7 (1648.9)	387.3 [709.8]	<0.001^*^
**Potassium, mg**	3640.5 (1446.5)	2406.3 [1885.6]	0.012^†^
**Zinc, mg**	11.8 (3.9)	7.8 (4.0)	0.005^*^
**Vitamins**			
**Vitamin A**, μ**g RE**	1056.8 (612.5)	718.5 (488.4)	0.012†
**Retinol**, μ**g**	196.2 (105.4)	6.4 [11.1]	<0.001^*^
β**-Carotene**, μ**g**	3756.9 [3741.8]	2937.8 [2581.5]	0.794
**Vitamin B**_**1**_**, mg**	1.9 (0.7)	0.9	[0.5] 0.002^*^
**Vitamin B**_**2**_**, mg**	1.6 (0.7)	0.6 (0.3)	<0.001^*^
**Vitamin B**_**6**_**, mg**	2.5 (1.1)	1.8 [1.7]	0.163
**Folate**, μ**g**	374 (219.2)	320.7 (222)	0.098
**Niacin, mg**	20.3 (8.2)	11.8 (4.4)	0.001^*^
**Vitamin C, mg**	245.4 (160.6)	124.7 [66.1]	0.094
**Vitamin E, mg**	15.2 (6.6)	8.2 (3.3)	0.001^*^

Data are presented as the mean (standard deviation) for normally distributed variables or as the median [interquartile range] for non-normally distributed variables.

Statistical comparisons were performed using a paired *t* test^*^ when the normality assumption was met; otherwise, the Wilcoxon signed-rank test^†^ was employed.

The *p* < 0.05 considered statistically significant.

RE, retinol equivalents.

**Fig. 1:**
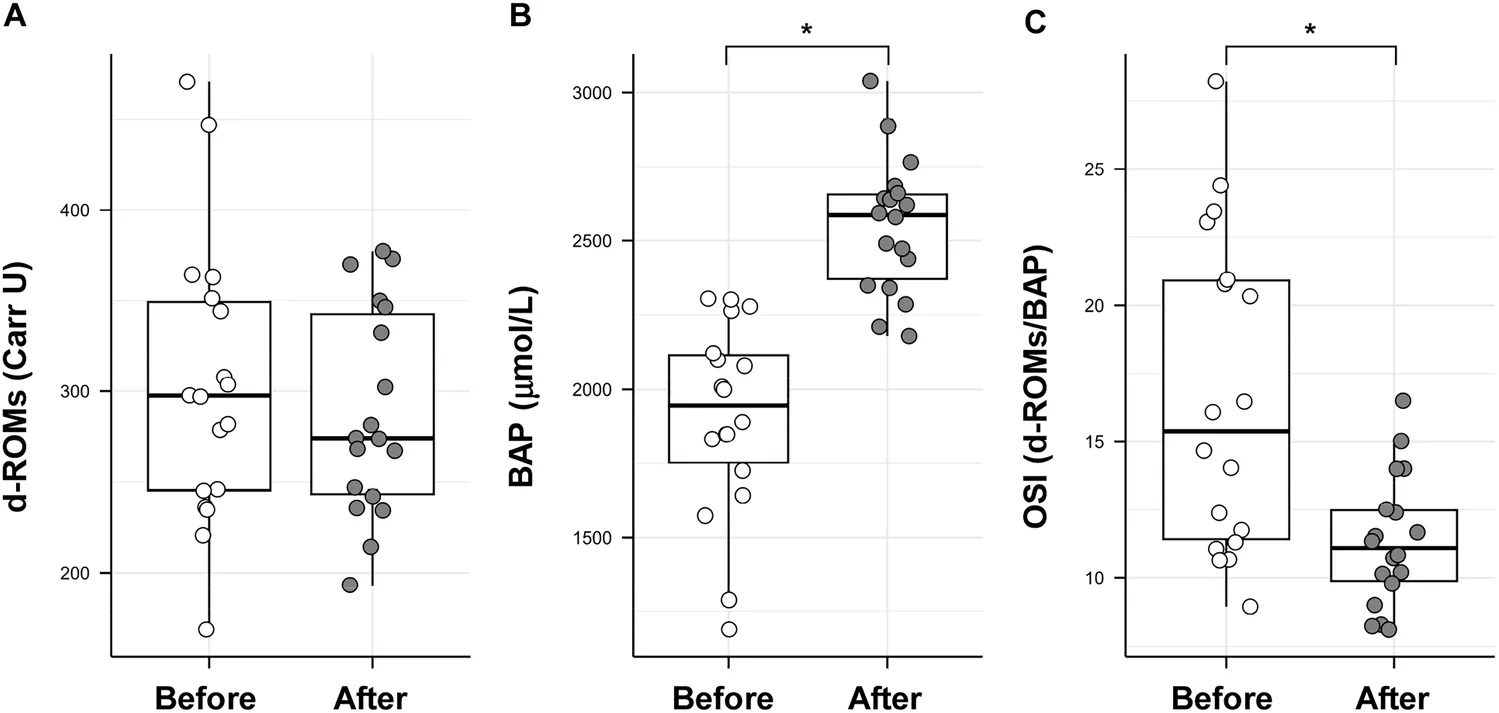
Antioxidant capacity following the 8-week plant-based dietary intervention. **A** d-ROMs, **B** BAP, and **C** OSI. The d-ROMs are expressed in arbitrary Carr U, where 1 Carr U corresponds to an oxidizing power of 0.08 mg H_2_O_2_/dL. The asterisk indicates statistical significance (^*^p < 0.05) determined by a paired *t* test when the normality assumption was met; otherwise, the Wilcoxon signed-rank test was used. d-ROMs Diacron-reactive oxygen metabolites, Carr U Carratelli units, BAP biological antioxidant potential, OSI oxidative stress index.

Serum d-ROMs levels decreased following the intervention (Fig. 1A), corresponding to increased antioxidant-rich vegetable and fruit consumption. The BAP concentration increased significantly from 1905.3 ± 328.3 to 2549.4 ± 228.1 μmol/L (*p* < 0.001; Fig. 1B). Notably, a BAP level >2200 μmol/L is considered indicative of adequate antioxidant capacity, suggesting that a plant-based diet effectively enhances systemic antioxidant defense. The OSI used to assess the balance between oxidative burden and antioxidant defense significantly decreased to 0.11 (*p* < 0.001; Fig. 1C).

**Fig. 2:**
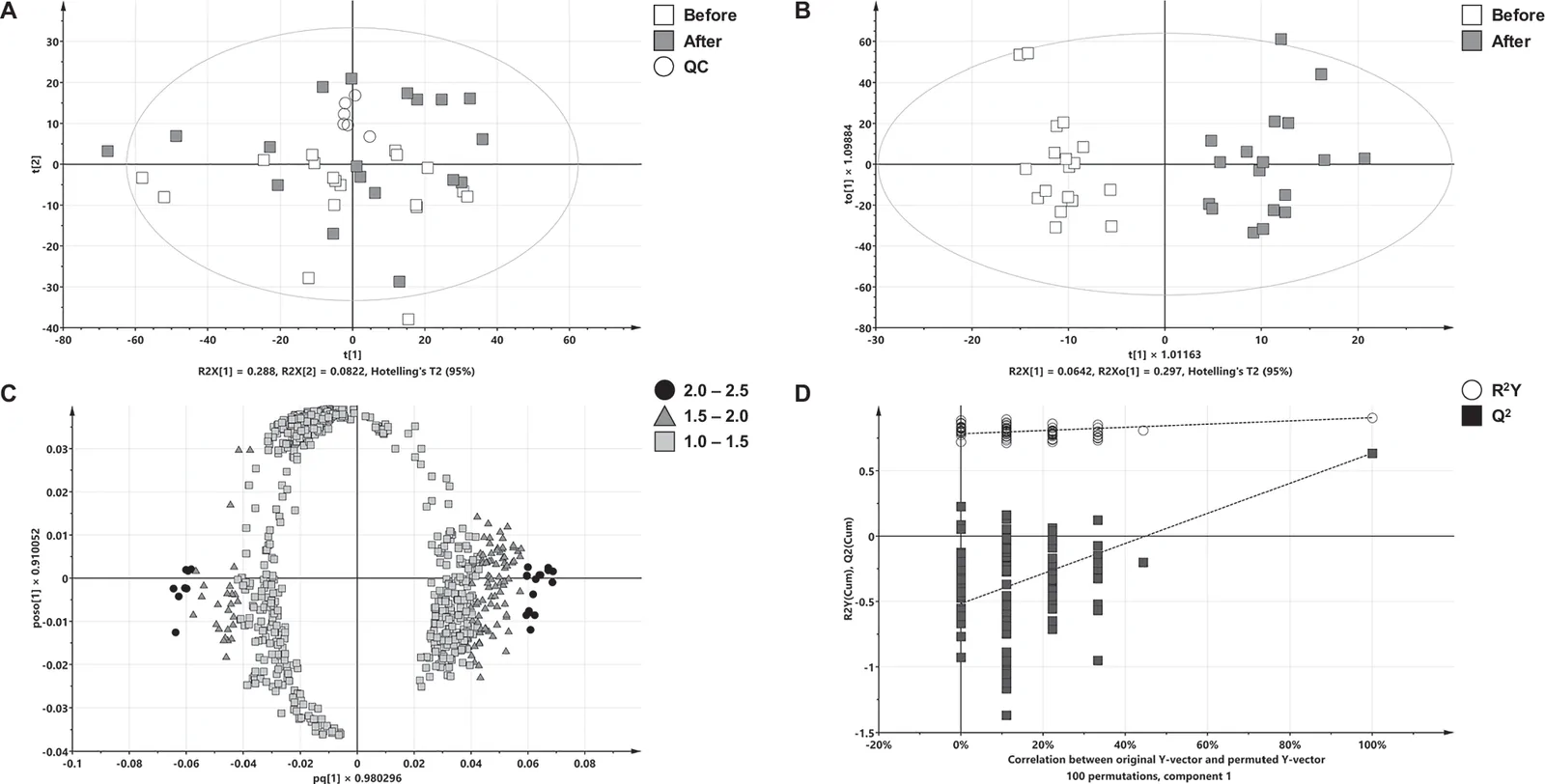
Multivariate analysis of untargeted urinary metabolomics data obtained using GC-MS. **A** The PCA plot demonstrates tight clustering of the QC samples, indicating high reproducibility during the analysis. **B** The OPLS-DA score plot reveals a clear distinction between samples collected before and after the plant-based dietary intervention. **C** The loading plot of X variables highlights those with a VIP ≥ 1.0. Features with VIP values between 1.0 and 1.5, between 1.5 and 2.0, and ≥2.0 are emphasized. **D** The validation plot of the OPLS-DA model reveals that the y-intercept of R^2^Y and Q^2^ was 0.783 and -0.517, respectively.

Pooled QC samples were analyzed alongside experimental samples. The relative standard deviation of peak intensities for the internal standard in the QC samples was <20%. Reliability was further supported by QC sample clustering in the PCA score plot (Fig. 2A). The OPLS-DA model demonstrated a clear separation between samples collected before and after the intervention of a plant-based diet (R^2^Y = 0.906, Q^2^ = 0.635; Fig. 2B). The number of features with a VIP value ≥ 1.0 was 835 (Fig. 2C). To assess the validity of the OPLS-DA model, internal validation using a permutation test was conducted, yielding R^2^Y and Q^2^ y-intercept values of 0.783 and –0.517, respectively (Fig. 2D).

**Fig. 3:**
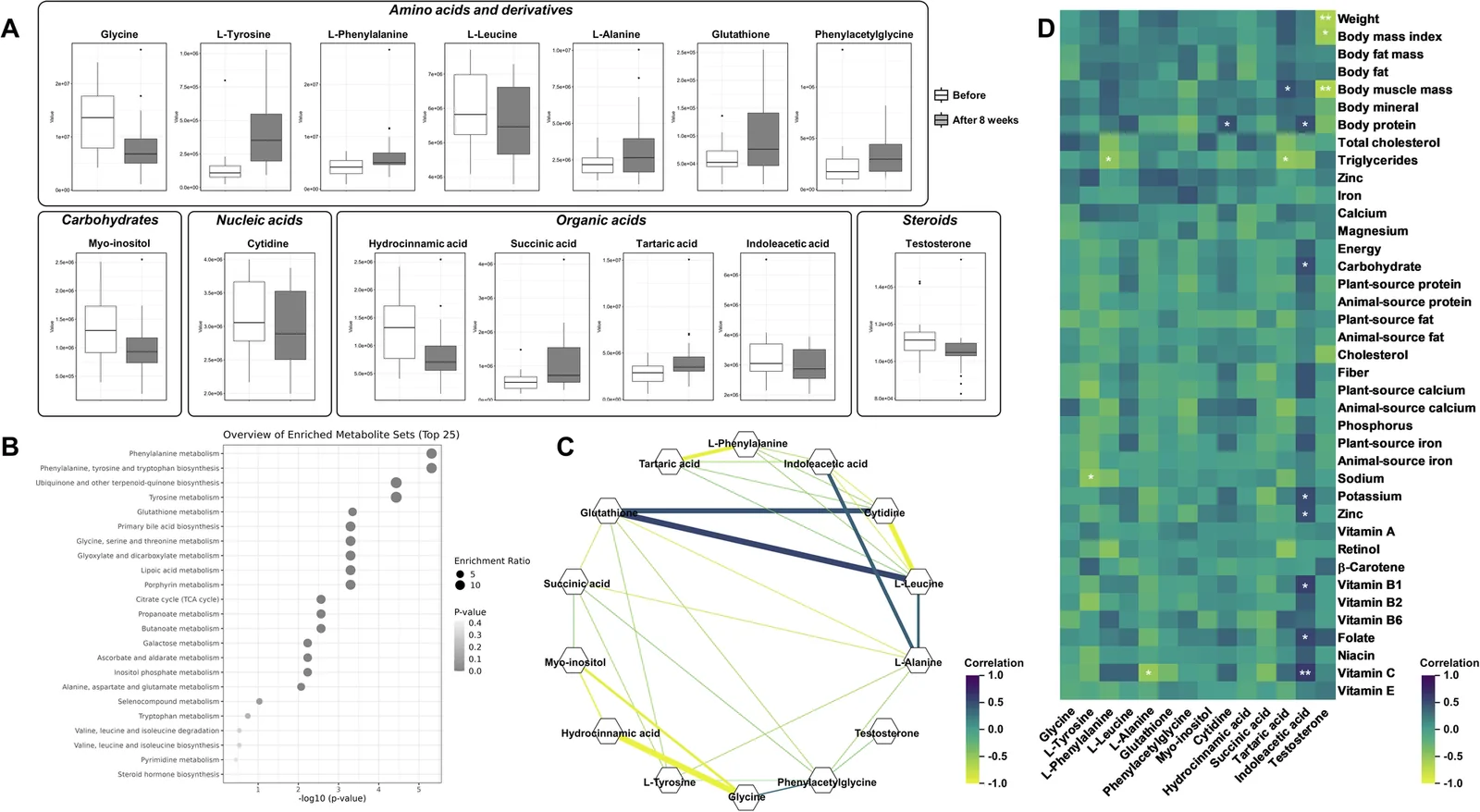
Metabolomic profiling and clinical correlation of the differential metabolites. **A** Box and whisker plots illustrating the 14 metabolites that were significantly altered following the 8-week plant-based dietary intervention. The box plots represent the interquartile range, encompassing 50% of the relative intensity of each metabolite. The whiskers indicate the maximum and minimum values of relative intensity. **B** Pathway analysis utilizing the KEGG database. **C** Metabolites-metabolites correlation analysis. The red and blue lines indicate positive and negative correlations, respectively. The thickness of each line corresponds to the strength of the correlation. **D** Metabolites-clinical/nutritional variables correlation analysis. Asterisks indicate statistically significant correlations (^*^p < 0.05 and ^**^p < 0.01) as determined by Pearson’s or Spearman’s correlation analysis, depending on the normality of the data distribution. Color intensity represents the correlation coefficient from –1.0 (purple) to +1.0 (yellow) using a colorblind-friendly viridis palette.

Thirty-three metabolites were annotated (Supplementary Table 3). Among them, 14 satisfied both criteria (VIP ≥ 1.0 and *p* < 0.05) and were considered significantly altered (Fig. 3A). These metabolites underwent pathway enrichment analysis, revealing several key pathways, such as phenylalanine, tyrosine, and tryptophan biosynthesis and metabolism; ubiquinone and other terpenoid-quinone biosynthesis; and glutathione metabolism (Fig. 3B). A correlation analysis of these 14 metabolites was conducted to explore their interrelationships (Fig. 3C). Hydrocinnamic acid and myo-inositol, as well as cytidine and L-leucine, were positively correlated, while glutathione exhibited strong negative correlations with L-leucine and L-alanine. Among the significant metabolites, four—glycine, hydrocinnamic acid, succinic acid, and tartaric acid—were identified with Level 1 confidence, as defined by the Metabolomics Standards Initiative [23], based on direct comparison of retention times and MS spectra with authentic standards analyzed under identical GC–MS conditions. Further correlation analysis explored potential associations modulated by dietary factors between metabolites and metadata, including clinical and biochemical parameters (Fig. 3D). L-Tyrosine, L-phenylalanine, and L-alanine demonstrated positive correlations with sodium, triglycerides, and vitamin C, respectively. Tartaric acid was positively correlated with triglycerides but negatively correlated with body muscle mass. Conversely, indoleacetic acid (IAA) displayed negative correlations with body protein and several nutritional parameters, including carbohydrates, potassium, zinc, vitamin B1, folate, and vitamin C. Testosterone exhibited significantly positive correlations with both body weight and muscle mass (all *p* < 0.001). As testosterone is a key sex hormone with potential sex-specific metabolic effects, sex-stratified subgroup analyses were conducted. Urinary testosterone levels significantly decreased in girls (*p* = 0.046) but not in boys (*p* = 0.182; Supplementary Fig. 1A). Testosterone showed a strong positive correlation with body weight (*p* = 0.001; Supplementary Fig. 1B) only in girls.

## Discussion

In this study, the 8-week plant-based dietary intervention was implemented for Korean adolescents. The intervention elicited notable improvements in clinical parameters. In parallel, plasma antioxidant capacity significantly increased, as evidenced by elevated BAP levels and reduced OSI, suggesting an enhanced systemic antioxidant defense. Multivariate analysis of urinary metabolite profiles revealed a separation between the samples collected before and after the intervention, indicating systemic metabolic changes in response to the dietary shift. Several key metabolites and pathways were identified, highlighting the broad impact of plant-based diets on adolescent metabolism.

The macronutrient distribution changed markedly during the intervention, with carbohydrates accounting for 84% of total energy intake. Although this proportion may appear high, it primarily reflects a relative increase due to reductions in fat and protein intake rather than excessive carbohydrate consumption. This pattern aligns with the characteristics of a plant-based diet emphasizing whole grains, vegetables, and fruits, which typically provide a higher proportion of carbohydrates and lower intakes of protein and fat compared to omnivorous diets [24, 25]. Importantly, in our study, the carbohydrates mainly originated from brown rice-based whole grains, providing complex carbohydrates and dietary fiber rather than refined starches or sugars, indicating an improvement in carbohydrate quality. Notably, the absolute carbohydrate intake decreased to 388 g/day, comparable to the national average of 343 g/day for Korean adolescents aged 15–18 years, based on the Korea National Health and Nutrition Examination Survey, 2010–2012 [26]. This suggests that the plant-based diet supplied sufficient energy and macronutrients to support normal growth and physiological needs in adolescents.

The intervention significantly reduced body weight, BMI, and body fat mass, indicating improved metabolic health. These findings suggest that even short-term dietary interventions can physiologically benefit adolescents, consistent with previous findings wherein vegetarian diets proved more effective than non-vegetarian diets for short-term weight loss [27]. The changes also reflect a moderated risk of future cardiometabolic disease, corroborating the role of plant-based dietary patterns in early prevention strategies [28]. Decreased animal-based fat consumption and increased fiber-rich plant food intake likely improved lipid metabolism and energy balance [29]. Moreover, sodium intake significantly decreased owing to reduced processed food consumption [30]. Although no mineral deficiencies were detected during the 8-week intervention, the overall intake of several minerals diminished. While these reductions did not cause clinically significant deficiencies, trace minerals are crucial for growth and development during adolescence For instance, calcium is essential for bone mineralization and the attainment of peak bone mass, whereas zinc deficiency can impair immune function, muscle development, and protein synthesis [31, 32]. Mineral absorption may also be reduced due to lower bioavailability caused by phytic acid, a unique natural compound found in many plant-based foods, which has a high affinity for chelating minerals [33]. Therefore, long-term adherence to plant-based diets may require dietary planning or supplementation to ensure adequate micronutrient intake. Details on bowel health and stress evaluation are in the supplementary information.

Plasma antioxidant capacity significantly improved, as evidenced by the increased BAP and decreased OSI values. These changes indicate systemic antioxidant defense upregulation, which is supported by a significant increase in urinary glutathione. These indicators suggest that a plant-based diet may offer systemic immune protection against oxidative stress by enhancing endogenous antioxidant mechanisms. Beyond clinical changes, gastrointestinal health and psychological stress were also investigated (Supplementary Table 4). Constipation was significantly alleviated, possibly because of decreased processed food consumption, while the prevalence of irritable bowel syndrome did not change significantly. In contrast to previous studies suggesting that plant-based diets may help alleviate psychological stress [34], stress scores slightly increased, along with a shift in the distribution of stress severity.

**Fig. 4:**
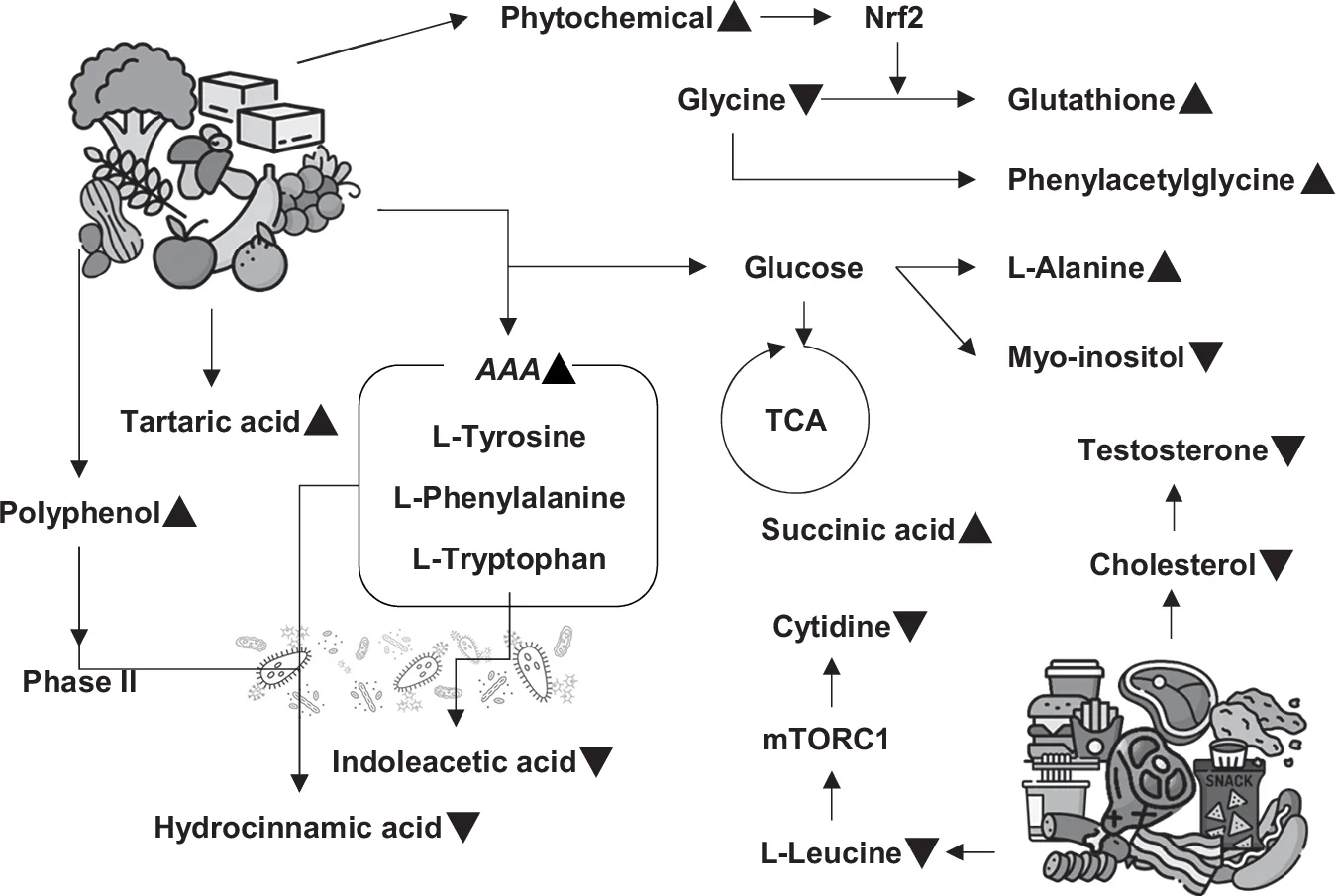
Proposed metabolic alterations associated with the plant-based dietary intervention in adolescents. The 8-week intervention led to an increased consumption of whole grains, vegetables, and fruits (left) alongside a concurrent decrease in the intake of animal-based and processed foods (right). These dietary shifts were associated with significant changes in urinary metabolites. The upward triangle (▲) and downward triangle (▼) indicate metabolites that increased and decreased, respectively, after the intervention compared to baseline. AAA, aromatic amino acids; TCA, tricarboxylic acid; Nrf-2, nuclear factor erythroid 2–related factor 2; mTORC1, mammalian target of rapamycin complex 1.

The coordinated changes in urinary metabolites provide a comprehensive understanding of how a plant-based diet can modulate metabolism (Fig. 4). Notable shifts in amino acids, particularly aromatic and branched-chain amino acids (BCAAs), were detected. L-Phenylalanine and L-tyrosine levels increased, and aromatic amino acid biosynthesis and metabolism were among the most significantly affected pathways. The intervention reduced L-leucine levels, aligning with previous findings wherein plant-based diets reduced serum BCAA concentrations [35]. Leucine activates the mammalian target of rapamycin complex 1 (mTORC1) pathway, promoting protein synthesis and cell growth [36]. The observed positive correlation between L-leucine and cytidine potentially aligns with previous findings wherein BCAAs proved necessary for nucleotide synthesis [37]. Additionally, increased complex-carbohydrate-rich whole grain intake may enhance pyruvate production and subsequent alanine synthesis through the glucose–alanine cycle [38]. This metabolic adaptation potentially explains the observed increase in L-alanine. Glycine is a key component of glutathione synthesis [39] and also participates in detoxification pathways via conjugation with benzoic acid, a gut microbial breakdown product of polyphenols [40]. The increased phenylacetylglycine levels demonstrated the contribution of glycine to phase II detoxification in the liver [41]. The reduced glycine levels suggest increased utilization for detoxification processes, potentially driven by a higher polyphenol-rich food intake. This highlights the relationship among dietary phytochemicals, gut microbial metabolism, and detoxification capacity. Furthermore, the inverse correlation between glutathione and L-leucine suggests that the body strengthens its antioxidant defense systems rather than growth-promoting pathways (*e.g*., the leucine-dependent mTORC1 pathway).

Succinic acid levels increased following the intervention. Elevated urinary succinic acid levels imply enhanced mitochondrial oxidative metabolism. Previous studies have revealed that elevated succinic acid levels may efficiently decrease adipose tissue mass and triglycerides in the liver and improve glucose tolerance [42]. Moreover, succinic acid can act as a signaling molecule, activating succinate receptor 1 on intestinal and immune cells [43], indicating that the plant-based dietary intervention might have promoted the beneficial modulation of immune function and energy.

Hydrocinnamic acid, derived from polyphenols or phenylalanine via microbial metabolism [40, 44], paradoxically decreased despite increased polyphenol consumption. This may reflect accelerated phase II transformations of polyphenols (e.g., glucuronidation and sulfation) [45]. Tartaric acid serves as a marker of fruit intake, since humans absorb and excrete it unchanged in urine [46]. Myo-inositol, a molecule involved in insulin signaling and glucose uptake [47], decreased after the intervention. Its co-variation with polyphenol-related metabolites suggests a potential interaction between glucose regulation and phytochemical metabolism [48]. Additionally, IAA, a microbial catabolite of tryptophan associated with proteolytic fermentation, exhibited negative correlations with multiple dietary and nutritional parameters, including protein, carbohydrates, zinc, and vitamin B1. This pattern is attributable to limited animal-based protein availability. Despite increased urinary L-tryptophan levels, IAA levels decreased. This suggests that tryptophan is metabolized through alternative metabolic pathways other than IAA production [49]. Although microbial profiling was not conducted, the observed metabolite patterns are consistent with modulation of the gut microbiome by dietary fiber [50].

Sex-stratified subgroup analysis revealed a significant decrease in testosterone levels in girls (*p* = 0.046). In girls, we observed a strong association between weight loss and testosterone reduction (*r* = 0.863, *p* = 0.001), suggesting that the plant-based diet intervention effectively improved hormonal profiles in girls. This finding is clinically meaningful, as elevated testosterone in adolescent girls is often associated with insulin resistance, increased risk of polycystic ovary syndrome and metabolic dysfunction [51]. Previous studies have shown that reductions in testosterone levels following weight loss are associated with enhanced insulin sensitivity and improved metabolomic profiles in women [52, 53]. Therefore, the significant decrease in testosterone levels in girls represents a favorable metabolic adaptation. In contrast, testosterone levels in boys showed a slight but non-significant decrease after the intervention (*p* = 0.182), with weaker correlations between testosterone changes and body composition parameters (*r* = 0.530 and *r* = 0.505, not significant). Overall, testosterone levels in boys remained stable, which is physiologically appropriate for adolescent male development, as testosterone plays essential roles in secondary sexual development, reproductive maturity, and attainment of muscle and peak bone mass during this period [54].

Despite these promising findings, this study has certain limitations. First, the sample size was small and the intervention period relatively short. Second, dietary assessments relied on self-reported questionnaires, which are prone to recall bias. Third, the non-analysis of vitamin B12, micronutrient levels, or gut microbiota limits the mechanistic interpretation of some findings. In addition, urinary metabolites represent a composite output of dietary, microbial, endogenous, and renal processes, including xenobiotic-derived compounds. Their concentrations are influenced by hydration, circadian rhythm, and renal clearance, making it difficult to distinguish specific metabolic sources. To minimize these confounding effects, medical history, medication use, smoking, and alcohol consumption of participants were carefully screened during recruitment, and all samples were collected as first-morning urine under fasting conditions to ensure comparability across participants. Given the exploratory nature of this untargeted metabolomics study, we did not apply multiple comparison correction. In exploratory metabolomics analyses without prespecified hypotheses, multiplicity adjustments are not strictly required [55], and a conventional *p* < 0.05 threshold is appropriate, particularly when findings are further evaluated using classification models [56]. In this study, we adopted a dual-filtering approach (*p* < 0.05 and VIP ≥ 1.0) to identify metabolites with both statistical significance and substantial contribution to group separation. Despite these limitations, this study provides important preliminary insights as the first urinary metabolomic investigation to examine the metabolic effects of a plant-based diet in Korean adolescents.

In conclusion, a plant-based diet elicits positive metabolic changes, as evidenced by increased whole grain, vegetable, and fruit consumption. These metabolite alterations are associated with body fat mass reductions and strongly correlated with antioxidant capacity improvements, underscoring the metabolic benefits of plant-based dietary patterns. Such findings provide valuable insights into how dietary patterns influence metabolic health in adolescents. Together with recent literature, these results corroborate the notion that even short-term dietary improvements can significantly enhance metabolic health. If these diets are sustainably integrated into school systems, they may have long-term positive impacts.

## Supplementary information

Supplementary information

## Data Availability

The datasets generated and/or analyzed during the current study are available from the corresponding author on reasonable request.
