# Supplementary material for: Urinary metabolomic profiling to identify the impact of the increased intake of whole grains, vegetables, and fruits in healthy adolescents
Source: Eur J Clin Nutr. 2026 Jul 29;80(10):1070–8. doi: 10.1038/s41430-026-01703-8 (PMC13690566; doi:10.1038/s41430-026-01703-8)
Supplement: Supplementary file 1 [file 41430_2026_1703_MOESM1_ESM.docx]

**Urinary metabolomic profiling to identify the impact of the increased intake of whole grains, vegetables, and fruits in healthy adolescents**

Mi-Ri Gwon^1,2†^, Jae Hwa Lee^1†^, Duk-Hee Lee^3^, Yoo Kyoung Park^4^, Soo-Jin Park^5^, Ji-Won Lee^6^, Hae Won Lee^1^, Sook Jin Seong^1,2*^, Young-Ran Yoon^1,2*^

^1^School of Medicine, Kyungpook National University and Department of Clinical Pharmacology and Therapeutics, Kyungpook National University Hospital, Daegu, Republic of Korea

^2^Clinical Omics Institute, School of Medicine, Kyungpook National University, Daegu, Republic of Korea

^3^Department of Preventive Medicine, School of Medicine, Kyungpook National University, Daegu, Republic of Korea

^4^Department of Medical Nutrition, Graduate School of East-West Medical Science, Kyung Hee University, Yongin, Republic of Korea

^5^Department of Histology and Anatomy, College of Korean Medicine, Daegu Haany University, Gyeongsan, Republic of Korea

^6^Department of Family Medicine, Severance Hospital, Yonsei University College of Medicine, Seoul, Republic of Korea

^†^These authors contributed equally to this work

Corresponding authors

E-mail: [yry@knu.ac.kr](mailto:yry@knu.ac.kr) (Y-R Yoon), wintersj@knu.ac.kr (SJ Seong)

**Supplementary Information**

**Supplementary materials and methods**

**Dietary intervention and assessment**

**Supplementary Table 1. Weekly meal plan for the plant-based diet**

| **Meal** | **Sun** | **Mon** | **Tue** | **Wed** | **Thu** | **Fri** | **Sat** |
| --- | --- | --- | --- | --- | --- | --- | --- |
| **Break-**  **fast** | •Brown rice •Bean sprout soup •Perilla leaf salad •Cucumber lettuce  •Kale wrap •Kimchi | •Brown rice  •Perilla seed mushroom hot pot  •Seasoned soft tofu  •Glass noodles with vegetables (japchae)  •Swiss chard namul  •Paprika salad | •Brown rice  •Soybean paste stew  •Stir-fried shiitake mushrooms  •Lettuce wrap  •Cucumber and chili peppers  •Kimchi | •Brown rice  •Soft tofu soup  •Cucumber–seaweed salad  •Braised konjac with peanuts  •Cabbage wrap  •Kimchi | •Brown rice  •Mallow soup  •Curry rice  •Seasoned grilled tofu  •Spinach namul  •Young radish kimchi | •Brown rice  •Mallow soup  •Seasoned burdock root  •Broccoli salad with pine nut sauce  •Cabbage wrap  •Kimchi | •Brown rice  •Spinach soybean paste soup  •Seasoned bean sprouts  •Seasoned eggplant  •Kelp wrap  •Young radish kimchi |
| **Lunch** | •Brown rice  •Shredded radish soup  •Steamed radish greens  •Sweet and sour chicken  •Seasoned chamnamul  •King oyster mushroom  •Fresh sweet cabbage kimchi | •Brown rice  •Hand-pulled sticky rice dough soup  •Young summer radish greens  •Red Chili Paste Pancake  •White kimchi | •Brown rice  •Fried tofu and crown daisy soup  •Seasoned soft tofu  •Fresh sweet cabbage kimchi  •Butterbur wrap  •Cubed radish kimchi | •Brown rice  •Oyster mushroom soup  •Seasoned bean sprouts  •Seasoned chamnamul  •Kelp wrap  •Kimchi | •Brown rice  • Crown daisy and tofu soup  •Seasoned stone crop  •Seasoned cucumber  •Stir-fried king oyster mushrooms and paprika  •Cabbage wrap  •Kimchi | •Brown rice  •Chard soup  •Water parsley and radish fresh salad  •Seasoned radish greens  •Stir-fried oyster mushrooms  •White kimchi | •Brown rice  •Bean sprout kimchi stew  •Seasoned water parsley and mung bean sprout  •Stir-fried button mushrooms  Braised walnuts and peanuts  Korean daisy kimchi |
| **Dinner** | •Bibimbap  •Sweet cabbage soup  •Grilled tofu  •Kimchi | •Brown rice  •Radish and green onion soup  •Seasoned wild chive and cucumber  •Stir-fried seaweed stems  •Zucchini pancake  •Kimchi | •Burdock root bibimbap  •Bean sprout and radish soup  •King oyster mushroom pancake •Tangerine  •Kimchi | •Brown rice  •Radish soup  •Seasoned fatsia shoots  •Braised lotus root  •Enoki mushroom vegetable salad  •Whole cabbage soybean paste | •Thistle bibimbap  •Tofu soybean paste soup  •Bellflower root salad with pine nut sauce  •Kimchi | •Brown rice  •Soybean paste soup  •Seasoned chwi namul  •Braised burdock root  •Angelica and wild ginseng salad  •Fresh cucumber •kimchi | •Brown rice  •Soft tofu soup  •Chicory salad  •Seasoned cucumber  •Seasoned Spinach namul  •Fresh seaweed |

Namul refers to lightly seasoned vegetables. Bibimbap is rice mixed with assorted vegetables and seasonings. Kimchi and white kimchi are fermented vegetable dishes; the latter is non-spicy.

**Supplementary Table 2. Selected food group of food frequency questionnaire for adolescents**

| **No.** | **Food group lists** | **Food (reference)** |
| --- | --- | --- |
| 1 | Rices | Plain rice, Rice with mixed grains, Rice with beans (210 g each) |
| 2 | One dishes | Bibimbab, Curry rice, Rolled rice with lever, Stuffed rice in fried soybean curd (each dish) |
| 3 | Ra-myeon | Ra-myon (1) |
| 4 | Noodles | Noodles, Mandu and soup, Chinese noodles (each bowl) |
| 5 | Breads | Bread (60 g), cake (50 g) |
| 6 | Hamburger, pizza, spaghetti | Hamburger (1), pizza (each slice), spaghetti (1) |
| 7 | Potatoes, sweet potatoes | Potatoes, sweet potatoes (100 g) |
| 8 | Meat - Soups | Beef-rib soup, Beat and bone soup, beef stew (each bowl) |
| 9 | Meat - Grilled | Grilled beef with seasoning, Stir-fried pork (each dish) |
| 10 | Meat - Fryings | Pork cutlet, Fried pork with sweet and sour sauce (each dish) |
| 11 | Pork, ham, sausages | Grilled pork belly (80 g), Ham (80 g), Sausage (80 g) |
| 12 | Fried chicken | Fried chicken (2 slices), Fried chicken with sweet and spicy sauce (4 slices) |
| 13 | Fish stew | Fish stew, Sea food stew (each bowl) |
| 14 | Fish cake | Fish paste (60 g) |
| 15 | Grilled, fried or griddled fish | Mackerel (70 g), fried yellow Croaker (small size), griddled pollack (4 pieces), Yellow-fin tuna canned (1/2 can) |
| 16 | Squid | Squid (1/2) |
| 17 | Anchovy, Dried icefish strip | Stir-fried anchovy, dried (1 Tbsp), Grilled dried icefish strip (2 slices) |
| 18 | Eggs | Egg (1), Quail's egg (5) |
| 19 | Tofu, Soy-sauce stew | Tofu (80 g), Soy-sauce stew (each bowl) |
| 20 | Kimchi | Kimchi, Cubed radish kimchi, Kimchi stew (60 g each) |
| 21 | Vegetable side dishes | Seasoned spinach, Seasoned bean sprouts cooked, Seasoned mushroom (60 g each) |
| 22 | Fresh fruit, fruit juice | Apple (1/2), Mandarin (1), Orange juice (1/2 C, 100 cc) |
| 23 | Cookie | Snack cookies (1/2 pack), Biscuit (5) |
| 24 | Milk, Yogurt, Soy milk | Milk, Yogurt, Soy milk (1 pack each) |
| 25 | Ice cream | Ice cream, Ice shabet (1 piece each) |

English names of listed food items was referred from Food values of portions commonly used, The Korean Nutrition Information Center, The Korean Nutrition Society (1998)

Each item’s consumption frequency was classified into nine levels: 3 times a day, 2 times a day, once a day, 5–6 times a week, 3–4 times a week, 1–2 times a week, 2–3 times a month, once a month, and rarely eat.

**Bowel health and stress assessment**

Bowel health was assessed using Rome II criteria for the diagnosis of functional constipation and irritable bowel syndrome (1). A participant was diagnosed with functional constipation if two or more of the six criteria were met within the preceding 3 months. Notably, irritable bowel syndrome was diagnosed when a participant experiences recurrent abdominal pain or discomfort for ≥3 days per month during the preceding 3 months, accompanied by two or more of the following criteria: a) improvement with defecation, b) onset associated with a change in stool frequency, and c) onset associated with a change in stool form. Stress was evaluated using the validated questionnaire specifically designed for adolescents (2). The questionnaire comprised 14 items covering academic stress, friendships, relationships with teachers and family, and self-perception of body image. Stress levels were assessed using the perceived stress scale (3).

**Chemicals and reagents for metabolomics profiling**

Methanol, heptane, and organic solvents (analytical and high-performance liquid chromatography grade) were procured from Merck (Darmstadt, Germany). Deionized water was obtained using Milli-Q^®^ and Elix^®^ systems (Millipore, Billerica, MA). Urease and methoxylamine hydrochloride were sourced from Sigma-Aldrich (St. Louis, MO). Glycine, hydrocinnamic acid, succinic acid, and tartaric acid were obtained from Fluka Chemical Corp. (Buchs, Switzerland). The internal standard, myristic acid (1,2,3-^13^C_3_, 99%), was purchased from Cambridge Isotope Laboratories, Inc. (Andover, MA). The silylation reagent, *N*-methyl-*N*-trifluorotrimethylsilyl acetamide with 1% trimethylchlorosilane, was acquired from Thermo Fisher Scientific Inc. (Waltham, MA).

**Supplementary results**

**Supplementary Table 3. Putative metabolite alterations following the plant-based dietary intervention**

| **VIP** | **Retention time** | **Metabolite** | **HMDB ID** | **Fold change** | ***p*** |
| --- | --- | --- | --- | --- | --- |
| **Amino acids** | | | | | |
| 2.17 | 21.1 | Glycine | HMDB00123 | 0.69 | 0.008 |
| 2.17 | 6.3 | L-Tyrosine | HMDB00158 | 4.59 | 0.006 |
| 1.41 | 40.5 | L-Phenylalanine | HMDB00159 | 2.15 | 0.037 |
| 1.40 | 6.1 | L-Tryptophan | HMDB00929 | 2.20 | 0.139 |
| 1.35 | 21.1 | L-Aspartic acid | HMDB00191 | 1.09 | 0.797 |
| 1.32 | 9.8 | L-Leucine | HMDB00687 | 0.94 | 0.014 |
| 1.19 | 10.5 | L-Alanine | HMDB00161 | 1.52 | 0.026 |
| 1.12 | 18.8 | L-Histidine | HMDB00177 | 0.98 | 0.279 |
| **Amino acid derivatives** | | | | | |
| 1.84 | 26.8 | Glutathione | HMDB00125 | 2.05 | 0.044 |
| 1.82 | 30.6 | Phenylacetylglycine | HMDB00821 | 2.15 | 0.013 |
| **Carbohydrates** | | | | | |
| 2.00 | 6.8 | Myo-inositol | HMDB00211 | 0.81 | 0.028 |
| 1.46 | 23.7 | Fructose 6-phosphate | HMDB00124 | 0.91 | 0.107 |
| 1.44 | 24.5 | Mannitol | HMDB00765 | 1.59 | 0.089 |
| 1.35 | 40.5 | D-Arabitol | HMDB00568 | 1.01 | 0.875 |
| 1.33 | 6.2 | D-Glucose | HMDB00122 | 0.98 | 0.296 |
| **Fatty alcohols** | | | | | |
| 1.05 | 11.1 | 1-Hexadecanol | HMDB03424 | 1.13 | 0.204 |
| **Nucleic acids** | | | | | |
| 1.40 | 27.7 | Deoxyuridine | HMDB00012 | 1.05 | 0.475 |
| 1.25 | 9.8 | Cytidine | HMDB00089 | 0.95 | 0.029 |
| 1.24 | 8.2 | Adenine | HMDB00034 | 0.96 | 0.164 |
| 1.21 | 16.8 | Uracil | HMDB00300 | 1.26 | 0.093 |
| **Organic acids** | | | | | |
| 2.07 | 22.4 | Hydrocinnamic acid | HMDB00764 | 0.74 | 0.013 |
| 1.74 | 11.9 | Succinic acid | HMDB00254 | 2.34 | 0.006 |
| 1.55 | 10.5 | Stearic acid | HMDB00827 | 0.81 | 0.055 |
| 1.42 | 11.0 | Tartaric acid | HMDB00956 | 1.97 | 0.033 |
| 1.40 | 11.0 | Citric acid | HMDB00094 | 1.59 | 0.112 |
| 1.32 | 9.8 | Indoleacetic acid | HMDB00197 | 0.92 | 0.009 |
| 1.16 | 35.1 | trans-Ferulic acid | HMDB00954 | 1.10 | 0.447 |
| 1.13 | 24.5 | Fumaric acid | HMDB00134 | 1.02 | 0.805 |
| **Steroids** | | | | | |
| 1.18 | 26.0 | Testosterone | HMDB00234 | 0.94 | 0.013 |
| 1.11 | 39.4 | Epiandrosterone | HMDB00365 | 1.18 | 0.722 |

Statistical comparisons were performed using a paired *t*-test when the normality assumption was satisfied; otherwise, the Wilcoxon signed-rank test was employed.

VIP, variable importance in projection; HMDB, human metabolome database

**Supplementary Figure 1. Sex-stratified analyses for testosterone.** (A) Box and whisker plot illustrating testosterone in boys (*n*=8) and girls (*n*=10). Testosterone levels significantly decreased in girls (*p*=0.046), but not in boys (*p*=0.182) after the intervention. (B) Correlation analysis between testosterone, body weight and body muscle mass for boys (left panel) and girls (right panel). Numbers represent correlation coefficients determined by Pearson or Spearman's correlation analysis according to normality distribution. Asterisks indicate statistical significance (^*^*p*<0.05, ^**^*p*<0.01, and ^***^*p*<0.001). Color intensity represents the correlation coefficient from –1.0 (purple) to +1.0 (yellow) using a colorblind-friendly viridis palette.

Following the 8-week plant-based dietary intervention, the baseline scores of parameters representing quality of life were compared (Supplementary Table 4). The intervention significantly improved bowel movement, with the proportion of participants experiencing constipation decreasing from 83% to 11%. Additionally, none of the participants satisfied the diagnostic criteria for irritable bowel syndrome; however, the overall distribution of subtypes did not change significantly. After the intervention, the perceived stress scale score slightly increased from 28.6 ± 7.7 to 33.4 ± 7.0, although this difference was not statistically significant. Similarly, the distribution of stress levels before and after the intervention was not statistically significant.

**Supplementary Table 4. Effects of the plant-based diet on quality of life**

| **Variable** | **Baseline** | | **After 8 weeks** | | ***p*** |
| --- | --- | --- | --- | --- | --- |
| **Constipation** |  |  |  |  | 0.004 |
| **No, *n* (%)** | 3 | (17) | 16 | (89) |  |
| **Yes, *n* (%)** | 15 | (83) | 2 | (11) |  |
| **Irritable bowel syndrome** |  |  |  |  | 0.198 |
| **No, *n* (%)** | 14 | (78) | 18 | (100) |  |
| **Constipation predominant, *n* (%)** | 1 | (6) | 0 | (0) |  |
| **Diarrhea predominant, *n* (%)** | 1 | (6) | 0 | (0) |  |
| **Unsubtyped irritable bowel syndrome, *n* (%)** | 2 | (11) | 0 | (0) |  |
| **Perceived stress scale** | 28.6 | (7.7) | 33.4 | (7.0) | 0.105 |
| **Stress** |  |  |  |  | 0.639 |
| **Light, *n* (%)** | 0 | (0) | 0 | (0) |  |
| **Moderate, *n* (%)** | 1 | (6) | 2 | (11) |  |
| **High, *n* (%)** | 10 | (56) | 4 | (22) |  |
| **Severe, *n* (%)** | 7 | (39) | 12 | (67) |  |

Statistical comparisons were performed using a paired *t*-test for continuous variables, while McNemar’s or Bowker’s test were employed for binary paired and multi-category paired variables.

**References**

1.	Longstreth GF, Thompson WG, Chey WD, Houghton LA, Mearin F, Spiller RC. Functional bowel disorders. Gastroenterology. 2006; 130:1480-1491.

2.	Lee K. Stress, social support, and behavior problems of adolescents. Unpublished master’s thesis, Chonnam National University, Gwangju. 1997.

3.	Cohen S, Kamarck T, Mermelstein R. A global measure of perceived stress. J Health Soc Behav. 1983; 24:385-396.
